# Supplementary material for: Evaluation of Wild Peanut Species and Their Allotetraploids for Resistance against Thrips and Thrips-Transmitted Tomato Spotted Wilt Orthotospovirus (TSWV)
Source: Pathogens. 2023 Aug 28;12(9):1102. doi: 10.3390/pathogens12091102 (PMC10536083; doi:10.3390/pathogens12091102)
Supplement: Supplementary file 1 [file pathogens-12-01102-s001.zip › pathogens-2543345-supplementary.pdf]

pathogens

Supplementary file

Evaluation of Wild Peanut Species and their Allotetraploids for Resistance Against Thrips and Thrips-Transmitted Tomato Spotted Wilt Orthotospovirus (TSWV)

Yi-Ju Chen[1], Sudeep Pandey[1], Michael Catto[2], Soraya Leal-Bertioli[3,4], Mark R. Abney[5], Sudeep Bag[6], Mark Hopkins[4], Albert Culbreath[6] and Rajagopalbabu Srinivasan[1*]

1 Department of Entomology, University of Georgia, Griffin, GA 30223, USA; yijuchen@uga.edu, su-deep.pandey@uga.edu
2 Department of Entomology, University of Georgia, Athens, GA 30602, USA; mac65630@uga.edu
3 Department of Plant Pathology, University of Georgia, Athens, GA 30602, U.S.A; sorayab@uga.edu
4 Institute of Plant Breeding, Genetics and Genomics, University of Georgia, Athens, GA 30602, U.S.A mark.hopkins@uga.edu
5 Department of Entomology, University of Georgia, Tifton, GA 31794; U.S.A.; mrabney@uga.edu
6 Department of Plant Pathology, University of Georgia, Tifton, GA 31793, USA; sudeepbag@uga.edu
* Correspondence: babusri@uga.edu

Materials and Methods

S1. Thrips feeding damage index

Scale (0-3)	0	1	2	3
Feeding scar intensity of individual leaflet	**None** 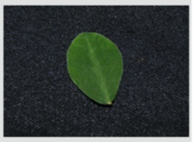	**<25%** 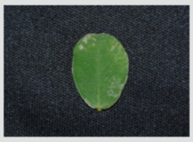	**25-50%** 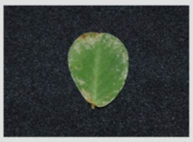	**>50%** 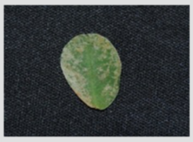

Figure S1. Thrips feeding scar intensity scale 0-3.
